# Dataset of Cavendish banana transcriptome in response to chitosan coating application

**DOI:** 10.1016/j.dib.2020.105337

**Published:** 2020-02-26

**Authors:** Fenny Martha Dwivany, Husna Nugrahapraja, Eiichiro Fukusaki, Sastia Prama Putri, Cindy Novianti, Septhy Kusuma Radjasa, Tessa Fauziah, Lutfi Dewi Nirmala Sari

**Affiliations:** a School of Life Sciences and Technology, Institut Teknologi Bandung, Bandung, 40132, Indonesia; b Biosciences and Biotechnology Research Center, Institut Teknologi Bandung, Bandung, 40132, Indonesia; c Bali International Research Center for Banana, Badung, Bali, 80361, Indonesia; d Department of Biotechnology, Graduate School of Engineering, Osaka University, 2-1 Yamadaoka, Suita, Osaka, 565-0871, Japan

**Keywords:** Edible coating, Fruit ripening, Postharvest, RNA-Seq

## Abstract

Banana is a climacteric fruit and its ripening process is greatly influenced by presence of ethylene. This physiological climacteric characteristic of banana fruit leads to a fast ripening and a short shelf-life. Application of edible coating such as chitosan aims to prolong fruit shelf life. The knowledge on gene expression will help to understand the fruit ripening process itself and chitosan effect on global gene expression. Global gene expression data of chitosan treated and control of Cavendish banana during fruit ripening were provided. Total RNA was isolated from banana pulp for differential gene expression analysis. The RNA-sequencing generated ranged from 16,155,947 to 23,587,110 total reads, with 75.8%–83.8% of reads were mapped against the genome reference. In total, 33,797–35,944 transcripts were detected. The transcriptomics data discussed in this publication are accessible through NCBI's Gene Expression Omnibus with GEO Series accession number GSE139457. These data provide information to identify candidate genes involved in fruit ripening in response to chitosan coating to design a better banana postharvest management.

**Table:** Specification Table

Subject Area	Biological Sciences
Specific subject area	Fruit ripening
Type of data	Transcriptomics data (abundance measurements generated from RNA-seq data)
How data were acquired	Illumina HiSeq. 2000 platform
Data format	Raw: fastq.gz files Processed Data: Tab-delimited text files with FPKM values
Parameters for data collection	Transcriptomics of Cavendish banana pulp from uncoated and chitosan coated conditions
Description of data collection	Total RNA was derived from pulp of uncoated and chitosan coated Cavendish banana and was sequenced by using Illumina Hiseq 2000
Data source location	Bandung, West Java, Indonesia (6°53′28.9″S 107°36′38.3″E)
Data accessibility	NCBI's Gene Expression Omnibus (GEO) with GEO series accession number GSE139457https://www.ncbi.nlm.nih.gov/geo/query/acc.cgi?acc=GSE139457 Raw data are available at NCBI's Sequence Read Archive (SRA) database (accession number SRP227182) https://trace.ncbi.nlm.nih.gov/Traces/sra/?study=SRP227182
Related research article	Lustriane, F.M. Dwivany, V. Suendo, M. Reza, Effect of chitosan and chitosan nanoparticles on post harvest quality on banana fruits, J Plant Biotechnol 43 (2018) 36–44. https://doi.org/10.5010/JPB.2018.45.1.036 [1]

**Table:** 

**Value of the Data** • These data provide the first information of global gene expression in response to chitosan coating during banana ripening. • These data are crucial to identify candidate genes involved in fruit ripening in response to chitosan coating. • These data will be useful to design a better management of banana postharvest.

## 1 Data description

This paper provides transcriptomics data of uncoated (control) and 1.25% chitosan-coated banana fruit to understand the delay in fruit ripening mechanism by chitosan coating. Previous study by Lustriane et al. [1] showed that chitosan was able to delay banana fruit ripening. The changes in global transcriptome during the course of ripening of uncoated and chitosan-coated banana were evaluated in order to obtain a better understanding on the mechanisms involved in ripening delay upon chitosan treatment and applied to design a better postharvest management. The transcriptomics dataset files generated from the 12 sets of *Musa acuminata* pulp (uncoated and chitosan coated fruit) have been deposited to Gene Expression Omnibus (GEO) NCBI database [2]. The RNA-seq raw data are available at NCBI's Sequence Read Archive (SRA) database. Description of the materials, total RNA extraction, sequencing and transcriptome construction are given in the next section. The transcriptomics dataset statistics could be seen in Table 1, Table 2.

**Table 1:** Output statistics of the transcriptome from day 1 control (1K), day 1 chitosan-coated (1A), day 7 control (7K), and day 7 chitosan-coated (7A) of *Musa acuminata* (AAA Group) ‘Cavendish’ fruit in each replicate (replicate a, b, and c) of paired-end expreriment.

No.	Samples ID	Total Reads	Total Nucleotides	GC Percentage
1.	1K_a_1.fastq.gz	16,710,129	1,687,723,029	51
2.	1K_a_2.fastq.gz	16,710,129	1,687,723,029	51
3.	1K_b_1.fastq.gz	18,934,874	1,912,422,274	51
4.	1K_b_2.fastq.gz	18,934,874	1,912,422,274	51
5.	1K_c_1.fastq.gz	18,217,207	1,839,937,907	49
6.	1K_c_2.fastq.gz	18,217,207	1,839,937.907	48
7.	1A_a_1.fastq.gz	18,726,200	1,891,346,200	49
8.	1A_a_2.fastq.gz	18,726,200	1.891.346.200	49
9.	1A_b_1.fastq.gz	20,302,208	2,050,523,008	49
10.	1A_b_2.fastq.gz	20,302,208	2,050,523,008	49
11.	1A_c_1.fastq.gz	16,155,947	1,631,750,647	51
12.	1A_c_2.fastq.gz	16,155,947	1,631,750,647	51
13.	7K_a_1.fastq.gz	23,587,110	2,382,298,110	50
14.	7K_a_2.fastq.gz	23,587,110	2,382,298,110	50
15.	7K_b_1.fastq.gz	16,699,855	1,686,685,355	51
16.	7K_b_2.fastq.gz	16,699,855	1,686,685,355	51
17.	7K_c_1.fastq.gz	18,626,323	1,881,258,623	48
18.	7K_c_2.fastq.gz	18,626,323	1,881,258,623	48
19.	7A_a_1.fastq.gz	18,734,174	1,892,151,574	50
20.	7A_a_2.fastq.gz	18,734,174	1,892,151,574	50
21.	7A_b_1.fastq.gz	19,532,908	1,972,823,708	50
22.	7A_b_2.fastq.gz	19,532,908	1,972,823,708	50
23.	7A_c_1.fastq.gz	20,828,843	2,103,713,143	49
24.	7A_c_2.fastq.gz	20,828,843	2,103,713,143	49

∗All samples of RNA-seq raw data are available at NCBI's Sequence Read Archive (SRA) database with the accession number: SRP227182.

**Table 2:** Mapping result and transcripts detection of the transcriptome from day 1 control (1K), day 1 chitosan-coated (1A), day 7 control (7K), and day 7 chitosan-coated (7A) of *Musa acuminata* (AAA Group) ‘Cavendish’ fruit.

No.	Sample	Mapped Reads (%)	Transcripts Detected	Accession Number
1.	1K_a.txt	82.7	34,630	GSM4141871
2.	1K_b.txt	81.7	34,830	GSM4141872
3.	1K_c.txt	77.6	33,973	GSM4141873
4.	1A_a.txt	77.6	34,226	GSM4141874
5.	1A_b.txt	79.0	33,796	GSM4141875
6.	1A_c.txt	82.8	34,966	GSM4141876
7.	7K_a.txt	83.8	35,943	GSM4141877
8.	7K_b.txt	81.6	35,683	GSM4141878
9.	7K_c.txt	75.8	34,571	GSM4141879
10.	7A_a.txt	80.5	35,363	GSM4141880
11.	7A_b.txt	81.2	35,468	GSM4141881
12.	7A_c.txt	78.8	34,340	GSM4141882

## 2 Experimental design, materials, and methods

### 2.1 Plant material

Ethylene-treated banana fingers were used as samples for fruit ripening process. Three biological replicates of banana pulp samples were taken on 1st and 7th day from each untreated and 1.25% chitosan treated banana, as previously described by Pratiwi et al. [3], Lustriane et al. [1], Yamamoto et al. [4] and with modification.

### 2.2 RNA isolation

Total RNA was extracted from fruit pulp of each sample using Cordeiro's method [5]. The RNA concentration and quality were examined with NanoDrop spectrophotometer (Eppendorf BioSpectrometer® Kinetic) at 230, 260, and 280 as well as electrophoresis on 1.5% agarose gel. DNAseI kit from Thermo Scientific (Catalog Number: EN0521) was then used to purify RNA samples. The cDNA synthesis was then performed using the iScript™ cDNA Synthesis kit (Biorad Catalog Number: 170–8890) in thermal cycler (Applied Biosystem™ Veriti™ 96-Well Fast Thermal Cycler).

### 2.3 RNA library construction and sequencing

The TruSeq RNA Sample Prep KIT v2 was then used to construct RNA library from each sample and sequenced using Ilumina platform HiSeq 2000 with HCS V2.2 software.

### 2.4 Data analysis

Each sample was checked for quality control using *FastQC* program to examine low base score, adapter, and PCR contaminations in order to obtain clean reads [6]. Then, the clean reads were mapped against *Musa acuminata* DH Pahang Version 2 Genome Sequences (https://banana-genome-hub.southgreen.fr/sites/banana-genome-hub.southgreen.fr/files/data/fasta/version2/musa_acuminata_v2_pseudochromosome.fna) [7] using *TopHat* software [8,9] (Trapnell et al., 2009, 2012). Finally, aligned reads were quantified and normalized with FPKM value using *Cufflinks* V2.2.1 [9,10].

## Author contributions

**Fenny Martha Dwivany**: Conceptualization, Methodology, Validation, Writing (Original & Editing), Supervision. **Husna Nugrahapraja**: Resources, Validation, Analysis, Investigation, Data Curation, Writing (Original & Editing), Visualization. **Eiichiro Fukusaki**: Writing (Original & Editing). **Sastia Prama Putri**: Writing (Original & Editing). **Cindy Novianti**: Analysis, Investigation, Writing (Original & Editing), Visualization. **Septhy Kusuma Radjasa**: Analysis, Investigation, Writing (Original & Editing), Visualization. **Tessa Fauziah**: Analysis, Investigation, Writing (Original & Editing), Visualization. **Lutfi Dewi Nurmala Sari**: Analysis, Investigation, Writing (Original & Editing), Visualization.

## References

[1] Lustriane C., Dwivany F.M., Suendo V., Reza M. (2018). Effect of chitosan and chitosan nanoparticles on post harvest quality on banana fruits. J. Plant Biotechnol..

[2] Edgar R., Domrachev M., Lash A.E. (2002). Gene Expression Omnibus: NCBI gene expression and hybridization array data repository. Nucleic Acids Res..

[3] Pratiwi A.S., Dwivany F.M., Larasati D., Islamia H.C., Martien R. (2015). Effect of chitosan coating and bamboo FSC (Fruit Storage Chamber) to expand banana shelf life. AIP Conf. Proc..

[4] Yamamoto K., Amalia A., Putri S.P., Fukusaki E., Dwivany F.M. (2018). Expression analysis of 1-aminocyclopropane-1-carboxylic acid oxidase genes in chitosan-coated banana. HAYATI J Biosci..

[5] Cordeiro M.C.R., Silva M.S., Oliveira-Filh E.C., de Miranda Z.J.G., Aquino F.G., Fragoso R.R., Almeida J., Andrade L.R.M. (2008). Optimization of a method of total RNA extraction from Brazilian native plants rich in polyphenols and polysaccharides. IX Simposio Nacional Cerrado.

[6] Andrews S. (2010). FastQC: a Quality Control Tool for High Throughput Sequence Data. http://www.bioinformatics.babraham.ac.uk/projects/fastqc.

[7] Martin G., Baurens F., Droc G., Rouard M., Cenci A., Kilian A., Hastie A., Doležel J., Aury J., Alberti A., Carreel F., D'Hont A. (2016). Improvement of the banana “*Musa acuminata*” reference sequence using NGS data and semi-automated bioinformatics methods. BMC Genom..

[8] Trapnell C., Pachter L., Salzberg S.L. (2009). TopHat : discovering splice junctions with RNA-Seq. Bioinformatics.

[9] Trapnell C., Roberts A., Goff L., Pertea G., Kim D., Kelley D.R., Pimentel H., Salzberg S.L., Rinn J.L., Pachter L. (2012). Differential gene and transcript expression analysis of RNA-seq experiments with TopHat and Cufflinks. Nat. Protoc..

[10] Trapnell C., Williams B.A., Pertea G., Mortazavi A., Kwan G., van Baren M.J., Salzberg S.L., Wold B.J., Pachter L. (2010). Transcript assembly and quantification by RNA-Seq reveals unannotated transcripts and isoform switching during cell differentiation. Nat. Biotechnol..

